# One-hit wonders of genomic instability

**DOI:** 10.1186/1747-1028-5-15

**Published:** 2010-05-19

**Authors:** Alexander V Strunnikov

**Affiliations:** 1 Laboratory of Immunopathology, National Institute of Allergy and Infectious Diseases, National Institutes of Health, 5640 Fishers Lane, Room 1524, Rockville, MD 20852, USA

## Abstract

Recent data show that cells from many cancers exhibit massive chromosome instability. The traditional view is that the gradual accumulation of mutations in genes involved in transcriptional regulation and cell cycle controls results in tumor development. This, however, does not exclude the possibility that some mutations could be more potent than others in destabilizing the genome by targeting both chromosomal integrity and corresponding checkpoint mechanisms simultaneously. Three such examples of "single-hit" lesions potentially leading to heritable genome destabilization are discussed. They include: failure to release sister chromatid cohesion due to the incomplete proteolytic cleavage of cohesin; massive merotelic kinetochore misattachments upon condensin depletion; and chromosome under-replication. In all three cases, cells fail to detect potential chromosomal bridges before anaphase entry, indicating that there is a basic cell cycle requirement to maintain a degree of sister chromatid bridging that is not recognizable as chromosomal damage.

## Introduction

Due to recent advances in genome analysis, we now have access to genome-wide association studies in many cancer types [1,2], and, more importantly, to sequences [3,4] and chromosomal structures [5,6] of cancer genomes/exomes. These data show that DNA repeat instability and chromosome rearrangements in cancers, which were predicted and demonstrated in a number of early pioneer publications [7], are much more pervasive in occurrence and multi-faceted in nature than was previously anticipated. Furthermore, genome analyses of complex heritable diseases also indicate that multiple genomic changes must occur to attain the pathological phenotype [8,9]. Thus, studies of genome stability networks and of the mechanisms of chromosome destabilization have validated their vital importance for elucidating the origins of disease and for finding potential cures.

While the role of environmental damaging factors is well known in cancer and other complex diseases, the deregulation of internal cellular mechanisms that may interfere with genome stability is poorly understood. The fact that hundreds of complex syndromes are associated with chromosomal rearrangements including breaks, translocations, and tandem repeat instability, many of which occur at very specific hot spots of variability, indicates that disruption of global mechanisms of genetic homeostasis may be an underlying cause of such syndromes. Particularly, perturbations of high fidelity chromosome segregation during cell division may be involved. Thus, chromosome instability is apparently not just a signature (a "passenger") of many complex diseases, but also one of inherent causes ("drivers"). The severity and pathway specificity of the underlying mutation(s) in the genome homeostasis network therefore could be one of the key factors in the final clinical outcome of overt neoplasia.

A search for both universal and disease-specific mechanisms leading to multiple, rapidly-occurring genome-wide changes mandates the dissection of these mechanisms into specific biochemical/genetic pathways. While it is agreed that transcriptional deregulation is at the core of the final pathological pattern of most multigene diseases, chromosome rearrangements of a particular type, such as loss of heterozygocity (LOH) at different genomic regions, may make a very specific contribution to particular cases of aberrantly altered expression patterns. Behind such specificity are particular chromosomal zones that are destabilized if a given genome homeostasis pathway is disabled. For example, expansion of trinucleotide repeats, chromosomal translocations, and microsatellite instability all occur due to the dysfunction of distinct DNA housekeeping processes.

As a rule, cancer "tumor-suppressor" genes are defined based on the two-hit paradigm of Knudsen with a mutation in one allele accompanied by LOH [10]. However, a sizable fraction of genes involved in genome homeostasis are essential for cell viability, and thus cannot carry a hemizygous inactivating mutation. Instead, mutations of such genes could well be heterozygous but dominant. Indeed, chromosome instability traits in cancers were shown to be dominant [7]. Newly available data also show that cancer exomes carry a substantial load of heterozygous mutant alleles in genes responsible for chromosome stability and cell division (see below). Such mutations could be dominant-negative hypomorphs that contribute to the relaxation of genome integrity in cancers.

**Figure 1:**
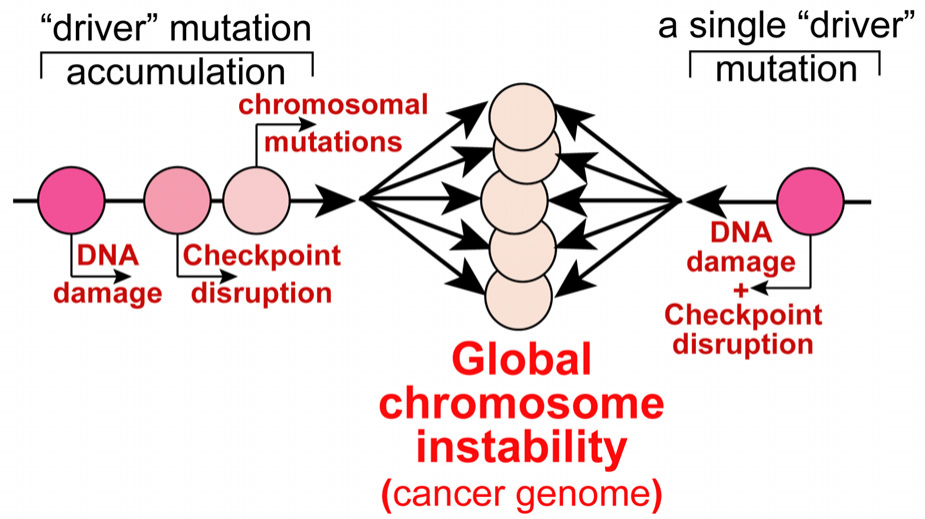
**Putative multi-step (left) and one-hit (right) genetic lesions leading to destabilization of cell cycle, chromosome integrity and genome homeostasis in cancer.** Circles represent genetic changes.

**Figure 2:**
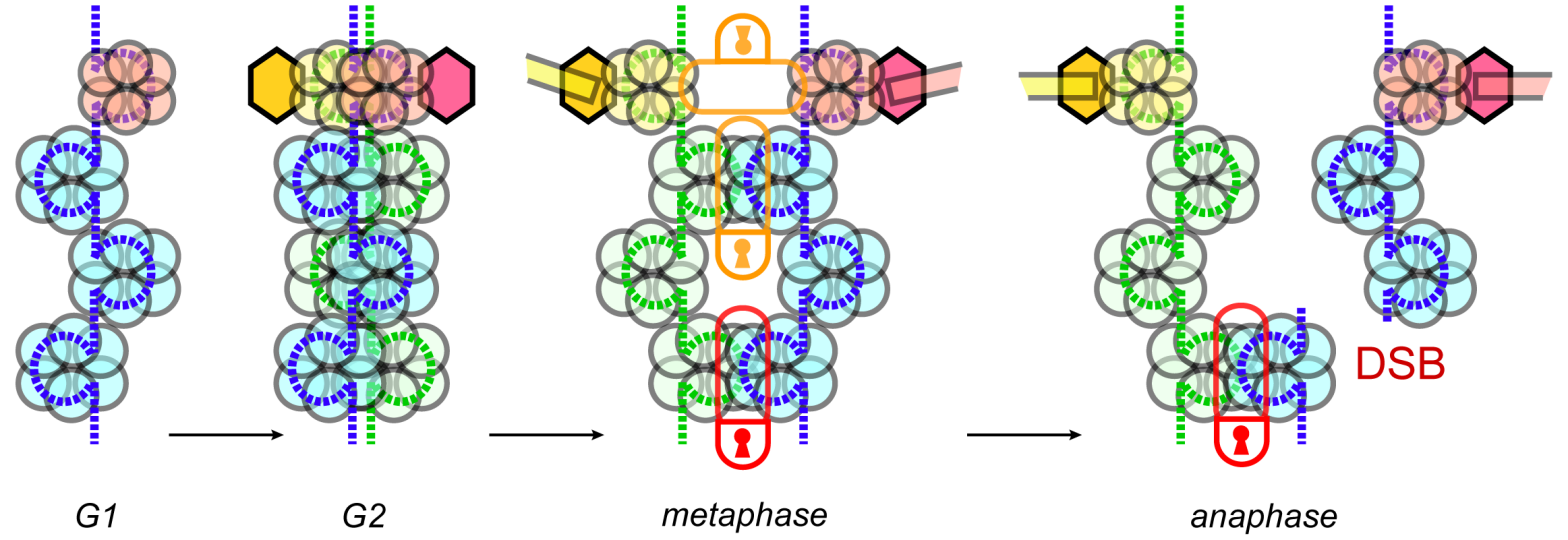
**Failure to resolve links/locks between sister chromatids leads to anaphase chromosomal breaks in higher eukaryotes**. Sister chromatids have differential color coding. DNA is represented by dotted lines, chromatin - by semitransparent circles, kinetochores - hexagons (with attached microtubules in mitosis), centromeric chromatin - semitransparent circles color-coordinated with corresponding kinetochores. Padlocks represent centromeric and arm sites where sister chromatids are attached to each other.

**Figure 3:**
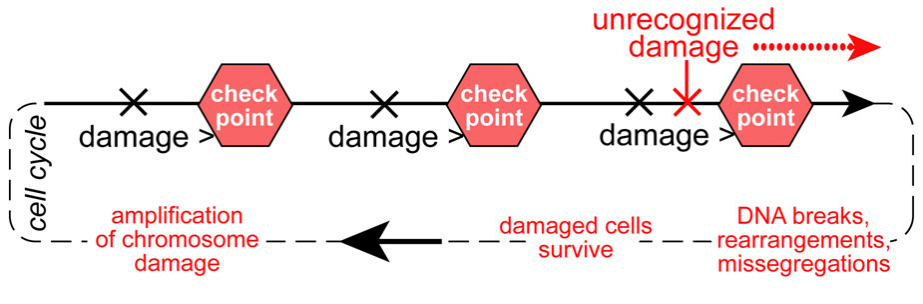
**Potential amplification of unrecognizable chromosomal damage**. The schematic depicts a hypothetical situation where specific chromosomal damage is not recognized by checkpoints, but it is not lethal, so that the accumulation of damage may occur in subsequent cell cycles.

Conventional wisdom suggests that two key changes are needed for sporadic genome reorganization: 1) a source of dramatically increased instability such as a mutation in a gene that results in global chromosome damage; and 2) the relaxation of checkpoint controls that normally detect and neutralize defects in DNA metabolism or integrity (Fig. 1). As a result of this two- or multi-step requirement for genome deregulation, the genetic homeostasis system is perceived as being very robust. However, this notion is challenged by accumulating contradictory evidence. Particularly, certain pathways/genes can be targeted with just a single mutagenic/inactivation event, which then generates both chromosomal damage and undermines cell division checkpoints. This subsequently may lead to rapidly accumulating and potentially heritable chromosomal mutations and rearrangements. Despite the differences in the underlying molecular mechanisms and the distinct chromosomal domains affected, one can identify distinct weak spots in cell division controls that are vulnerable to such defects. The mechanism common to the three cases discussed below is very specific. Namely, the chromosomal bridges (interlocked sister chromatid regions) are generated, but are left undetected by checkpoints. Then, when cells enter anaphase, these chromatid links may resolve via double-strand DNA breaks in late anaphase (Fig. 2). Breaks may result in LOH and may be liable to induce chromosome translocations [11]. Such a disruption of chromosomal integrity, when accompanied by the bypassing or undermining of cell cycle checks and balances, gives a powerful blow to genome integrity. It creates chromosome instability and potentially enables transmission of rearranged chromosomes to daughter cells. While in most cases such mutations are doomed to be lethal, one can imagine that damage would rapidly amplify in a cell lineage, if it is limited (e.g. the destabilizing mutation is hypomorphic) so that daughter cells are able to survive and propagate (Fig. 3). In this review, all three distinct examples of genetic lesions have the potential to create this type of instability. Genes and mutations of this kind are actually found among the heterozygous mutations associated with cancers.

### 1. Irresolvable cohesion between sister chromatids in anaphase. The cohesion release and separase pathway

Sister chromatids are held together from the first steps of replication until the moment when the signal is given to enter anaphase. At least two types of locks are present in sister chromatids: true topological interlocks via DNA catenation as a result of its replication, and the "cohesion", or "glue", links composed of proteins, best exemplified by the activity of the SMC complex called cohesin [12]. In the case of sister chromatid cohesion (SCC), both establishment and resolution are rather complex, dynamic, and highly regulated processes that differentially affect various chromosomal domains [13]. It is agreed that cohesion at the centromeres alone is essential for proper chromosome segregation in both mitosis and meiosis, because it is needed to establish bipolar attachment of sister kinetochores or homologous chromosomes, respectively. The role of cohesion at chromosome arms and telomeres is not fully understood, mostly because of the lack of tools to separate the functions of cohesin pools, this despite the fact that data point to substantial differences in these populations [14]. Furthermore, some reasonable assumptions were made that non-centromeric cohesin could have a mostly non-mitotic role, such as organizing chromosomal domain for DNA repair and/or gene regulation. This hypothetical function is supported by recent studies on non-dividing cells [15,16].

**Figure 4:**
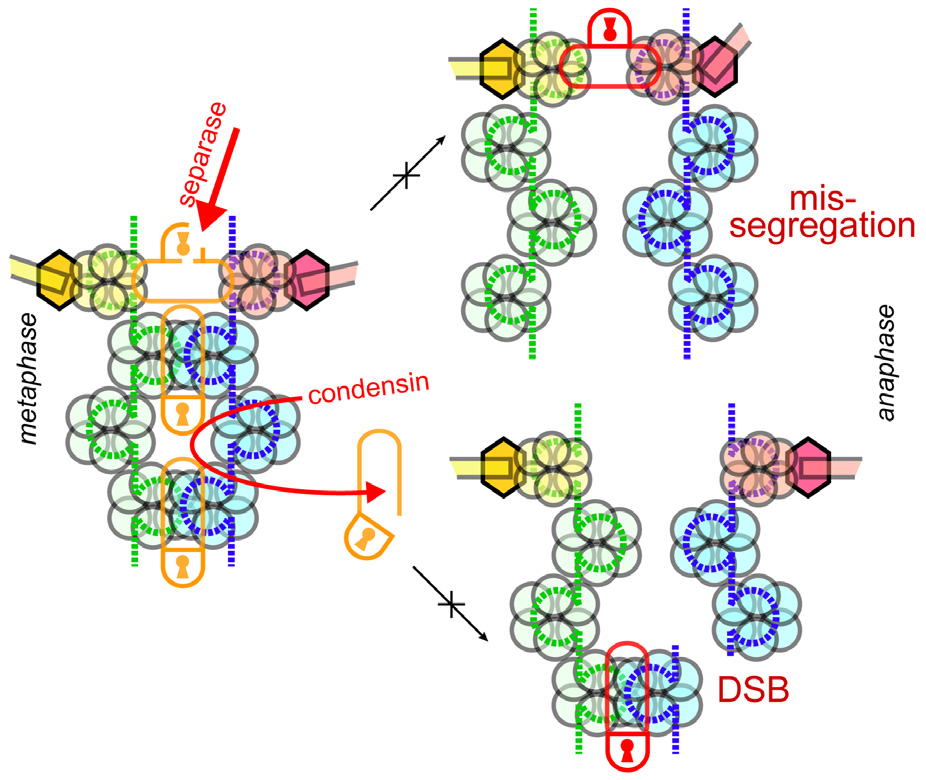
**Failure to release either centromeric or chromosomal arm cohesion leads to chromosome missegregation and breaks**. Shape and color coding as in Fig. 2. Separase inactivation results in massive missegregations, while condensin dysfunction leads to prominent DSB in anaphase.

Cohesin is proteolytically cleaved at the metaphase to anaphase transition, likely primarily at centromeres, releasing the interlocks between sister chromatids. If CDK1 is inactivated, this cleavage is sufficient to initiate chromosome segregation [17]. Thus, despite having other substrates, a site-specific proteolytic cleavage (Fig. 4) of the RAD21/SCC1/MCD1 subunit of the cohesin complex, is the main anaphase-triggering function of the protease termed separase [18]. Separase activity is tightly regulated in the cell cycle [19], presumably because its activity is highly undesirable at the centromeres prior to anaphase, when it may generate a loss of cohesion phenotype resulting in disorientation of sister centromeres (Fig. 4). Specifically, active spindle assembly checkpoint and DNA integrity checkpoints, either in unchallenged cells or upon damage, prevent separase activation, centromere separation, and anaphase entry by keeping the separase inhibitor and chaperone, PDS1/securin stable. The experimental artificial cleavage of centromeric cohesin rings indeed prematurely disrupts proper spindle attachment and bipolar orientation of sister chromatids in mitosis [18]. Furthermore, unscheduled cleavage of cohesin also results in detrimental consequences to non-diving cells [15,16], reflecting a more general role of cohesin in regulating genome structure.

In contrast to the premature loss of SCC that is effectively recognized by mitotic checkpoint, separase inactivation leads to complex chromosomal instability. This instability likely results from: 1) interlocks between sister chromatids generated by the failure to cleave even a small proportion of cohesin molecules (hence the dominant phenotype of noncleavable RAD21/SCC1/MCD1), and 2) invisibility of separase failures to the spindle checkpoint. Indeed, while activation of separase is under impressively multilayered control, it does not appear that there is any way for a cell to tell whether separase is fully active before initiation of anaphase. Furthermore, from the initial pioneering studies of *ESP1 *and *cut1 *genes [20,21], which encode separases in yeast, to subsequent extensive confirmations with separase knockdowns and noncleavable cohesin mutants in metazoa, it was evident that the checkpoint machinery fails to detect and react to damage associated with separase dysfunction. Mechanistically, failure to execute full activation of separase towards a cohesin target pool that is not removed by other mechanisms results universally in chromatids locked together at the centromeres so that proper segregation becomes highly problematic [22]. However, such a dramatic "lock" phenotype apparently does not prevent cells from going into cell division, neither in yeast [23], nor in Drosophila [24], human [25], or mouse [26] cells. As an example, separase-depleted HeLa cells that reach mitosis fail to resolve arms of sister chromatids, and their separation is perturbed in anaphase [25]. Mouse fibroblasts devoid of separase become highly aneuploid [26] because cytokinesis is not blocked, thereby indicating a profound failure of checkpoint controls. Essentially, eukaryotic cells lacking separase enter an aberrant "endocycle", where chromosome segregation is impeded but other cell cycle processes, including DNA replication, are allowed to continue. It remains to be determined if and what specific chromosome rearrangements may result from separase inactivation, or from a more specific defect - blocking the cleavage sites in the cohesin subunit SCC1/MCD1/RAD21 [27]. One can envision, however, that breaks in centromeric chromatin would be pervasive and thus cohesin cleavage defects may potentially be a factor contributing to Robertsonian fusions [28]. It is also notable that cancer cells that lost separase [27] can tolerate non-cleavable cohesin better then primary mouse cells [26]. One explanation for this would be the existence of additional separase targets; while an alternative, but not mutually exclusive, hypothesis is that separase dysfunction is tolerated in cancer as a result of an aberrant control of mitosis.

Noncleavable cohesin phenotype raises the question as to why are chromatids locked by unresolved SCC are not recognized by checkpoints? In the case of centromeric SCC, a recent study shows that cleavage of cohesin is sufficient to separate sister chromatids, but insufficient to segregate them, unless Cdk1 is inhibited independently [17]. This indicates that cohesin is the key generator of tension at sister centromeres, and therefore cells cannot recognize uncleaved cohesin as abnormal. Essentially, the microtubule occupancy and tension between the sister kinetochores, two key readouts for mitotic checkpoint, would remain unaffected if cohesin is uncleavable, because proteolysis of cohesin by separase occurs precisely at the metaphase-to-anaphase transition [27].

When cohesin is not removed from chromosomal arms in the course of the so-called prophase pathway [29], a simpler mechanism of checkpoint bypass is likely at play. When centromeric cohesion is resolved during the normal cell cycle, the mitotic checkpoint is inactivated [30] and therefore cannot sense potentially abnormal tension generated by the centromere-distant interlocks. Thus neither microtubule occupancy of kinetochores nor the tension components of mitotic checkpoint are equipped to deal with locks in chromosomal arms. Furthermore, triggering of DNA damage checkpoint is not possible not only because it is inactivated, but also because there is no molecular/recognizable damage before cells enter anaphase. One could also envision another, more specific, explanation of checkpoint failure when separase is inactivated. It is possible that the formation of an aberrantly stable complex between separase and noncleavable cohesin would delay anaphase to some degree, however such a delay would not be triggered if separase is absent altogether or a stable complex is not formed. No concrete experimental evidence of such a pathway exists. Furthermore, experiments showing that artificial cleavage of cohesin triggers anaphase [17] indicate that the topological integrity of the cohesin ring rather than some feedback signaling is solely responsible for the timing of anaphase initiation. Thus, the universal bypass of prolonged metaphase arrest upon separase inactivation is consistent with a simple structural explanation - locked sister chromatids represent the normal state of affairs up to the moment of segregation and, therefore, are not seen as defects.

It is not surprising that cohesin defects have been implicated in cancers as well as other syndromes [31-33], considering the involvement of this complex in many signaling pathways related to DNA damage and kinetochore function. Unfortunately, in most cases the depth of analysis was insufficient to indicate whether it is the premature loss of cohesion or the failure to release SCC that was predominately associated with the disease. As a result, it is currently not possible to tell whether potential "single hits" are among these mutations. For separase, both separase overexpression [34] and mutations [35] have been associated with tumorigenesis. In the latter case, one could safely hypothesize that it was the failure to release SCC and the ensuing checkpoint bypass that were the predominate cause of tumors. This could thus be the first direct experimental demonstration of a sustainable "single hit" tumorigenic mutation in metazoa (zebrafish). It is still unclear, however, what place such mutations may actually take in the oncogenic transformation process in humans, where tumorigenesis is evidently more complex. Are they are among driving mutations in tumors or are they involved in tumor progression or maintenance?

### 2. Merotelic kinetochores - interplay between Aurora B and condensins

Another possibility for how locks between sister chromatids can be carried into anaphase is the unresolved topological links between chromatids. For example, without type II topoisomerases, chromatids cannot segregate and may potentially break during mitosis. However, the very same activity of topoisomerase II is also required for transcription and DNA replication. Therefore, topoisomerase II-defective cells do not have the ability to bypass checkpoints because of the checkpoint-recognizable DNA damage that occurs prior to mitosis. While DSB do occur in topoisomerase II-inactivated cells, it is very likely that they are of non-mitotic origin, that is they correspond to the sites of incomplete topoisomerase reactions. In contrast, the SMC complex called condensin [36-39] has attracted considerable attention as the primary activity critical to removal of residual catenations [40] as well as any other possible interlocks between sister chromatids, including removing cohesin from chromosomal arms in prophase [29,41]. In vertebrates, there are two condensin complexes, condensin I and condensin II [42], which are differentially regulated [43]. Because condensin I and condensin II generally have an additive effect on chromosome segregation [44,45], condensin is referred to here as a single activity.

As a result of both condensin binding to DNA [46] and its enzymatic function that actively reconfigures supercoiled DNA when coupled to topoisomerase activity [47], sister chromatids are prepared for separation, with only a few impeding obstacles, before anaphase is triggered. Furthermore, condensin is required for the maintenance of condensed chromatids through metaphase and may have an additional compaction function in anaphase [48]. While the exact molecular nature of condensin activity in native chromatin is not known, the phenotypic characteristics of condensin inactivation in a variety of systems indicates that sister chromatids fail to disjoin, even if a reasonable degree of mitotic chromatin compaction is achieved [38]. Furthermore, studies of budding yeast conclusively demonstrated that an essential function of condensin is to separate sister chromatids into two distinct entities in metaphase, and to maintain this state through anaphase. Compaction is an apparent consequence of this key sister-sorting activity [37]. A specialized form of condensin activity in chromosome segregation involves partitioning of chromosomal regions that are still transcribed in mitosis, such as those in the nucleolus organizer locus (NOR, or rDNA) in budding yeast. There, condensin plays a key role in segregating the repeats that are actively transcribed by RNA Pol I, by virtue of acting on Pol I - silent rDNA repeats [49]. This epigenetically-controlled function of condensin apparently alleviates the negative effects of catenation and transcription-induced overwinding of rDNA, possibly by inducing positive supercoiling in this extremely busy region.

Following initial reports that suggested a specific requirement of condensin at centromeres [44,50-52], more recent studies have established that condensin has a definitive function there [45,53-55]. Furthermore, the molecular nature of condensin requirements specifically at centromeres is gradually being revealed. For example, the absence of condensin activity in budding yeast results in substantial separation of sister kinetochores in metaphase, while cells do maintain a robust prolonged arrest due to the activity of the mitotic checkpoint [53]. Analyses of potential checkpoint signals in that study clarified molecular mechanisms involved in condensin action at centromeres by documenting the loss of histone CENP-A from condensin-depleted centromeres. This finding indicated that condensin activity and CENP-A functions are intimately linked. Combined with the contemporaneous data on the unique structural features of centromeric chromatin, these results provide a strikingly fitting model for the specific requirement of condensin at centromeres. Namely, while condensin has been previously shown to positively supercoil circular naked DNA [56], reconstituted CENP-A chromatin was incidentally recently discovered to be positively coiled [57]. This raises the attractive possibility that condensin specifically facilitates positive coiling in centromeres to enable the formation of this specialized epigenetic domain.

**Figure 5:**
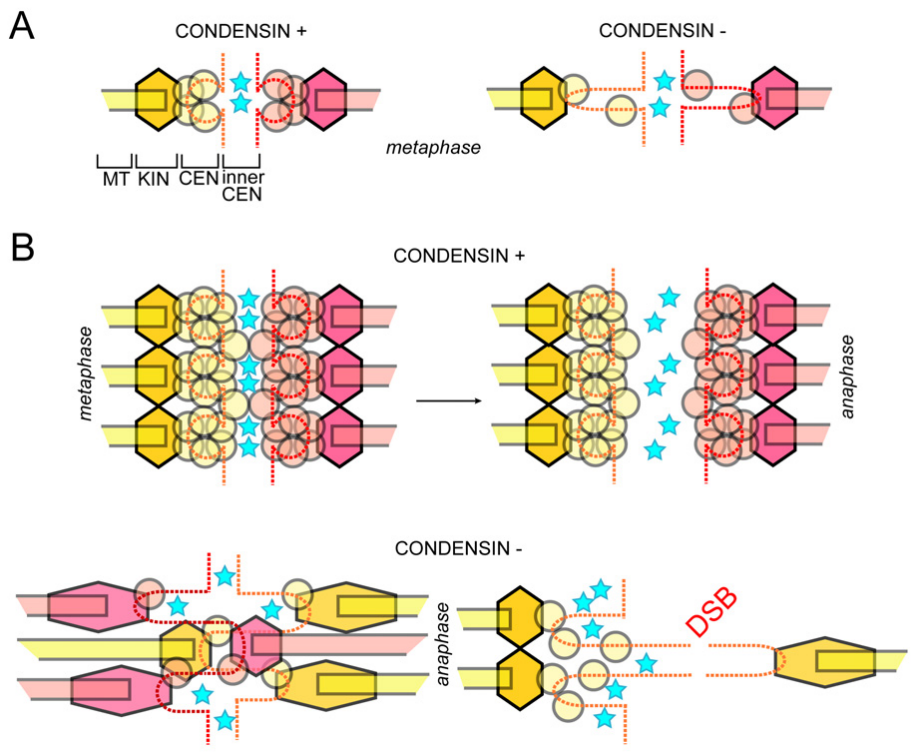
**Irreparable merotelic kinetochore misattachments upon condensin depletion from complex centromeres**. Shape and color coding generally follows Fig. 2, stars represent Aurora B molecules. (A) A 'simple' kinetochore, such as in budding yeast, with one (or few) microtubule-attachment modules, is not exposed to merotely upon decondensation of centromeric chromatin. However, the homologous Aurora B-centered machinery is present to correct syntelic misattachments. (B) Human centromeres and kinetochores suffer irreparable amount of merotely upon loss of condensin. Below: two scenarios that block repair of merotelic kinetochores deduced from analysis of condensin-depleted sister kinetochores in (*40*). Left - artificial balance of tension created by hyper-merotely; stretching both centromeres and kinetochores is illustrated. Right - centromere decondensation may result in individual microtubule-binding modules being removed from the zone where Aurora B is localized; a putative centromeric DNA break is depicted by a gap in the dotted line.

While condensin mutants in yeast were shown to induce mitotic checkpoint arrest [53], ample data suggest that condensin-depleted cells of metazoa do not enter a prolonged metaphase arrest, but instead proceed to anaphase with ensuing formation of chromosome bridges [58]. This dramatic contrast in mitotic checkpoint responses between yeast and higher eukaryotes prompted a recent investigation into the nature of centromere/kinetochore defects in veretebrates. It was shown that the mechanistic outcome of condensin inactivation is remarkably similar in yeast and human cells: centromeres are stretched in metaphase and CENP-A is substantially depleted [45]. The key difference between the two systems, the stretched appearance of kinetochores themselves in human cells, was strikingly similar to in vitro assembled *Xenopus *chromosomes [45]. This kinetochore stretching was nonetheless distinct from avian condensinless cells in which kinetochores appeared to be normal [54], indicating that condensation defects in centromeric chromatin do not necessary translate to defective kinetochores. This latter notion sets up a revealing paradigm: the biggest kinetochores, such as those in *X. laevis *and humans, evidently become defective upon the loss of condensin, while smaller kinetochores of chicken cells and single-microtubule kinetochores of budding yeast had proper morphology. The observed condensin knock down phenotype in *C. elegans *chromosomes [59], with their spread-out microtubule-attachment modules and accompanying pervasive merotely, strongly supports the idea that merotelic microtubule attachments are likely responsible for the deformed kinetochores in human cells depleted for condensin (Fig. 5). Indeed, a high degree of merotely was demonstrated in human cells depleted for condensin [45].

Merotelic attachments are thus becoming the key to explaining the fact that human cells do not maintain substantial metaphase arrest upon depletion of condensin. These cells enter anaphase because merotelic attachments are not recognized by checkpoints. The failure to detect all merotelic attachments even in wild type cells is evident from the fact that merotely is not completely corrected prior to anaphase, and few remaining mis-attachments are still seen in anaphase [60]. Two simple explanations for the spectacular failure of merotely correction in condensin depleted cells can be suggested. First, while occasional merotely in wild type cells generates an abnormal balance of tension at kinetochores with only a few microtubules misattached, the massive merotely in condensin-less kinetochores has a high probability of generating quasi-symmetric kinetochore-wide mis-attachments, which will effectively fool tension sensors (Fig. 5B). Second, the machinery that recognizes and corrects merotely, with the effector embodied by the Aurora B kinase, is thinly stretched over a larger area. This effectively separates the Aurora B pool from the kinetochore, which is apparently a physiological way to down-regulate Aurora B activity upon anaphase initiation [61]. The latter consideration prompted a study of the levels of centromeric Aurora B kinase activity in response to condensin depletion. Indeed, Aurora B phosphorylation of relevant substrates, such as CENP-A and MCAK, is dramatically reduced, with compelling evidence pointing to a correlation between the degree of stretching and the reduction of local Aurora B activity [45]. Furthermore, studies of a clear-cut model of merotelic attachments - drug-induced syntely, when all kinetochore microtubules are attached to one pole - demonstrated that condensin depletion and Aurora B inactivation are essentially epistatic. Namely, the inability of syntelic kinetochores to recover with the formation of bipolar attachments was indistinguishable between condensin-depleted, Aurora B-inactivated, and double-treated cells [45].

**Figure 6:**
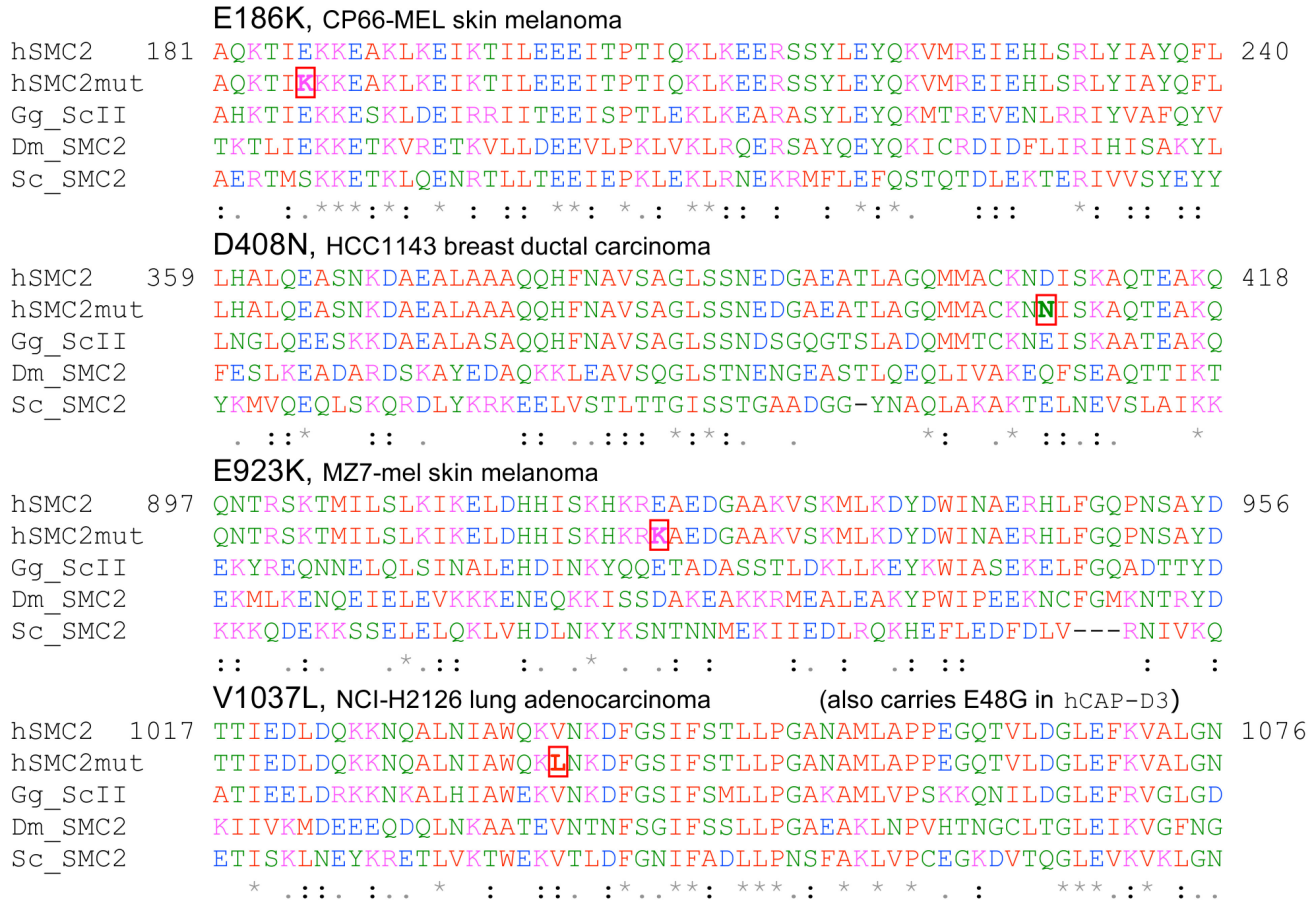
**Heterozygous mutation in the conserved regions of hSMC2 protein identified upon sequencing of cancer genomes**. Data is from COSMIC http://www.sanger.ac.uk/genetics/CGP/cosmic/. Only hSMC2 subunit mutations are shown for illustration. Other mutations are: NCAPD2 R698C, NCAPD3 E48G and L759L, NCAPG2 T246I. Alignment was generated and color coding was assigned by ClustalW software.

With respect to the initial premise of this review, genes encoding condensin subunits appear to clearly group with genes that we defined as one-hit drivers of genome destabilization. As was suggested above, mutations of these genes may be potentially associated with cancer genomes. Support for membership of condensin in this family has come from the fact that 8 (5%) of 159 sequenced cancer genomes and exomes in the COSMIC database contain missense mutations in condensin subunits (Fig. 6). It remains to be elucidated, however, whether they are passengers or true drivers of genome instability.

### 3. Incomplete chromosome replication

**Figure 7:**
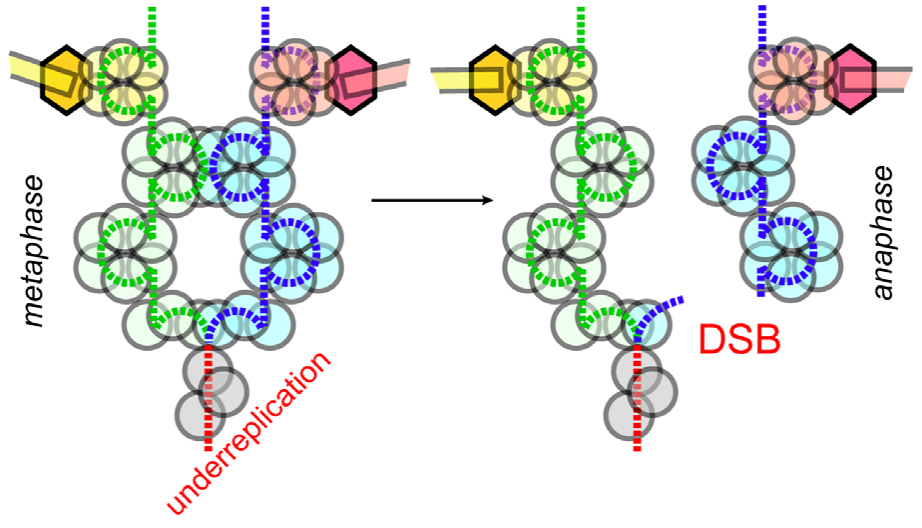
**DNA underreplication may result in chromosomal breaks in higher cells**. It is likely that yeast, due to their single-microtubule kinetochores, do not sustain substantial number of DSB as a result of chromatid bridging, opting for nondisjunction instead. It is likely, however, that human chromosomes would break, if underreplication zones are still present in anaphase.

Chromosomes of eukaryotic cells fully replicate strictly once per cell division, ensuring that a daughter cell receives only one complete set of genes - no more and no less. The licensing mechanisms that prevent over-replication by limiting origin firing to once per cell cycle were investigated in depth [62]. At the same time, mechanisms preventing chromosome under-replication are not well studied, especially in proliferating cells of higher eukaryotes. Remarkably little is known about the regulation of replication completion genome-wide and about the actual mechanism(s) of premature replication termination in cases where under-replication does occur as a part of normal developmental pathways [63-65]. Even less is known about the molecular basis of feedback signaling that tells a normal proliferating cell that DNA is fully replicated. It is obvious, however, that cells must receive some information about the length of S-phase [66] to ensure that replication is completed before mitosis, or at least before anaphase. If, however, an under-replicated chromosome enters anaphase, one may envision that an un-replicated zone larger than one Okazaki fragment would generate a physical link between sister chromatids, resulting in a chromosomal bridge and eventually a DSB (Fig. 7). A common reason for the failure to replicate parts of a genome is a stalled or collapsed replication fork, which may be processed into a DSB [67]. Such defects are recognized by DNA integrity checkpoints, particularly the intra-S phase checkpoint controlled by the ATR pathway that feeds information to the cell cycle in order to arrest cells in metaphase. This cell division arrest in response to a block in DNA replication provides a window of opportunity to repair the damaged fork. We still do not fully understand this pathway, as evidenced by the continuing discovery of additional control circuits [68,69]. Nevertheless, it is apparent that the mechanisms that prevent cell division when the DNA replication fork is damaged are multilayered and thus quite reliable. Therefore, ATR dysfunction has been associated with cancers [70]

At the same time, it appears that the signal of DNA replication termination genome-wide is not as robust as one might expect. For example, as was convincingly shown in budding yeast, DNA replication that is slowed but does not produce stalled forks or abnormal replication intermediates is not recognized by any known checkpoint. Slow moving forks could be generated by drugs, naturally-occurring slow replication zones, licensing of an inadequately low number of origins, and some specific but relatively minor defects in the replication machinery itself. For example, deletions and mutations of the carboxy-terminal domain in the main subunit of DNA polymerase epsilon POL2 resulted in under-replication unrecognized by checkpoints [71,72]. Furthermore, cells that failed to complete replication before entering anaphase suffered a dramatic loss of viability, apparently due to chromosome mis-segregation. While these initial publications proposed that this POL2 domain carries a special function interfacing DNA replication with the DNA integrity checkpoint, no separation of functions (checkpoint versus replication) was found. Furthermore, deletion mutants of the corresponding *S. pombe *gene, *cdc20*, did maintain a functional checkpoint [73]. *cdc20 *mutants, however, were shown to have a very slow DNA replication [73]. These results could therefore be interpreted in a completely different light, namely that slow DNA replication does not in general generate the types of DNA structures and/or signals that are able to activate the S-phase or DNA damage checkpoints.

Substantial support for this hypothesis was obtained upon analysis of the *sic1 *deletion in budding yeast. SIC1, a universal stoichiometric inhibitor of CDKs, is also required for setting up replication origins in G1 [74]. When SIC1 protein is absent, cells use fewer early origins and have larger replicons as a result. Consequentially, S-phase is extended but apparently not sufficiently to finish replication of the whole genome before entering anaphase. A substantial number of chromosome breaks and rearrangements follow [75]. Incidentally, no obvious defects in replication fork structure were identified in *sic1 *mutants, indicating that two putative prerequisites for bypassing the detection of under-replication took place: slow genome-wide replication with structurally normal replication forks.

Recently, another striking example of incomplete replication undetected by checkpoints was uncovered during studies in budding yeast of the mechanistic basis for anaphase chromatid non-disjunction in *cdc14 *mutants. In budding yeast, CDC14 is a multifunctional protein phosphatase that is essential for anaphase progression and exit from mitosis [76-78]. It is also a component of the DNA damage response in mammals [79,80]. In yeast, *cdc14 *mutants have a "classic" chromosomal bridge phenotype, with telomeres and the nucleolar organizer failing to disjoin. Even though conditional *cdc14 *mutations induce a tight late anaphase arrest due to the *CDC14 *requirement for exit from mitosis, the cells have very low viability when returned to permissive temperature, an indication of massive damage that is irreparable in anaphase. Extensive new data indicate that DNA under-replication is the essence of this damage as well as is the chief cause of chromosome mis-segregation in *cdc14 *mutants [81]. While early origins fire on time in CDC14-deficient cells and S-phase is not visibly extended, up to 10% of yeast genome is not replicated by the time *cdc14 *mutants enter anaphase. Especially affected are regions that normally replicate from late firing origins. Investigation of the mechanisms underlying this abnormality showed that the core defect appears to be linked to a substantial decrease in the amount of replication proteins in the nucleus, causing them to become a limiting factor for timely genome replication [81]. This deficiency in replication factors is due to nuclear import defects of two kinds. First, CDC14-dephosphorylation mediated import of SWI6, a transcription factor needed for late G1 transcription of many genes encoding replication proteins, is disrupted in the preceding mitosis [81,82]. Second, a more direct and prolonged importin-mediated defect occurs, targeting replication factors regulated at the level of nuclear entry, such as RFA2 subunit of replication protein A (RPA) complex [81]. As a result of these defects, DNA replication initiates normally from early origins, but apparently uses up all replication factors so that they are unavailable to initiate replication at late origins. Because the time of late origin firing normally precedes completion of replication from early ones, most of the genome has to be replicated from early origins. Similarly to *sic1 *mutants, analyses of the physical structure of replication forks in *cdc14 *mutants did not reveal any abnormalities except for a minor elongation lag, a feature consistent with RPA insufficiency. Thus, it is likely that slow moving replication itself results in incomplete genome replication being unrecognized by checkpoints.

One could only speculate as to why the whole genome cannot be replicated from the early origins in *cdc14 *mutants. It appears to be counter-intuitive, because CDC14 actually inactivates the anaphase-inhibiting phosphorylations generated by the S-phase-specific CDKs [83]. While one cannot exclude the possibility that CDC14 is also a component of the elusive signaling pathway that tells cells when replication is complete, this would require a very complex model, since CDC14 is likely to be active only in anaphase [84]. An alternative hypothesis suggested that the termination of DNA replication is controlled by the actual time cells spend in S-phase and G2, rather than by a specific signaling circuit [75]. The sum of the present knowledge suggests, however, that this is unlikely, mainly because cell cycle timing is highly variable in being dependent on environmental conditions and tissue specificity. Another hypothetical possibility is that completion of DNA replication may need to be coordinated with other processes that utilize CDC14 and facilitate finalizing of replication: 1) downregulation/repression of transcription in mitosis [49,85,86]; and 2) binding of condensin to DNA replication end-zones [87]. In case of *cdc14 *mutants, both of these processes are notably disrupted in the rDNA cluster; however a genome-wide investigation of a possible correlation between these processes has not been conducted. This hypothesis would require the assumption that CDC14 activity is not fully restricted to anaphase, that the final stages of DNA replication overlap with mitosis, or that these processes are preset in the preceding mitosis, all of which are rather controversial.

The failure of DNA integrity checkpoints to detect under-replication in *cdc14 *mutants is especially puzzling, considering the high degree of damage. A simple explanation is that a massive nucleo-cytoplasmic protein imbalance throws off the corresponding signaling mechanism. For example, the bulk of RFA2 was not phosphorylated by ATR/MEC1 in response to DNA damage in *cdc14 *cells [81]. Another possibility is that completion of replication in wild type cells is a normal biochemical function of a different process that occurs by default in mitosis and is thus is not monitored by checkpoints. For example, mitosis-specific condensin loading may facilitate the completion of DNA replication by virtue of repressing transcription and removing cohesin. Condensin recruitment to sites of replication termination was indeed demonstrated by a genome-wide ChIP-chip analysis [87]. There is also some circumstantial evidence suggesting that termination of DNA replication could indeed be related to mitosis, at least in some cell types. While under-replication has not been reported in proliferating cells of metazoa, it is apparent in tissues with developmentally-induced chromosome amplifications. These include fly nurse cells and larval tissues [63], placental trophoblasts in mammals [88], and multiple tissues in plants [89]. All these tissues without mitotic division replicate DNA via endocycle and coincidentally never replicate the complete genome [63]. The existing studies on endocycles did not address the nature of such under-replication, however they strongly suggest that the key to sensing under-replication is associated with regulatory cascades of mitosis itself, not just with APC oscillation [90].

Some more exotic explanations for the apparent lack of an identifiable signal for replication termination could be based on topological and structural chromosomal constraints instead of signaling cascades. For example, no special feedback signaling may be needed for the end of replication, if centromeres were to finish replication last, generating an automatic safety mechanism preventing the segregation of un-replicated chromosomes. This idea was discounted based on the demonstration of early replication of some centromeres in budding yeast [91]. However, several yeast centromeres coincide with DNA replication termini [92]. Furthermore some recent publications directly link centromere replication to DNA damage response. For example, centromeres fail to replicate when DNA damage checkpoint is compromised and cells are transiently exposed to replication inhibitors [93]. This indicates that, at least in a cell cycle challenged by DNA damage, the abovementioned safety mechanism may be in play. This study also established that the ATR pathway somehow controls centromere under-replication. In general, some still uncharacterized cell cycle-related functions of ATR in cells not challenged by DNA damage are probably the best candidates for feedback signaling of DNA replication completion. In this case, one must anticipate that slowed DNA replication will to be accompanied by the loss of some ATR targets that are used as a readout by the hypothetical S-phase completion signaling pathway. This is likely the case in *cdc14 *and *pol2 *mutants; however the role of losing individual ATR phosphorylation sites in general checkpoint responses remains to be investigated.

### Conclusion. Biological rationale for ignoring potential chromosome bridges: the price for speed and complexity

As discussed previously, cell division mechanisms in general and genome integrity specifically are surprisingly vulnerable to defects that reveal themselves only in anaphase as exemplified by chromosomal bridges/locks. Both DNA damage and mitotic checkpoints are inactivated in anaphase, so that even theoretically they could neither stop chromosome segregation in its tracks nor pull back to the metaphase. The so-called NoCut checkpoint, which involves Aurora B function [94], also cannot eliminate all anaphase chromosomal breaks, as it is active only after chromatid bridges, a precondition for breaks, have occurred. In fact, the NoCut checkpoint may even facilitate the transmission of rearranged chromosomes to daughter cells by virtue of providing the time necessary to heal the broken ends of chromosomes thereby enabling survival of cells that sustained structural chromosomal rearrangements.

The key problem is then why is such a lax quality-control of chromosomes for potential anaphase bridges maintained by natural selection? Providing a general answer to this question is seemingly impossible without invoking a multilayered weakness originating from the compelling need to keep sister chromatids together and then to separate them at once (the "price of speed"). Hence, it is easy to generalize why chromosome interlocks are not recognized as damage at metaphase; it is because there is no actual DNA or kinetochore/microtubule damage before chromosomes start moving to the poles. It is much more difficult to answer why there is no efficient "intra-anaphase checkpoint" to deal with the chromosomal bridge problem. The apparent technical impossibility to pullback the runaway train of anaphase chromosome segregation to a metaphase-like state for repairs is likely to be at the core of this weakness. This basic biological inability to hold/reverse anaphase once it has started may be due to the very biochemical basis that enables both high speed and synchrony of chromosome segregation: the regulation of anaphase by protein degradation and proteolytic cleavage such as in the cases of cohesin, securin, B-cyclins and other molecules that are destroyed just before and during anaphase. To deal with this challenging task, eukaryotic cells developed a way to compact and separate their sister chromatids ahead of time, long before anaphase, due to a consorted action of condensins, topoisomerases, and other proteins via the process rather superficially designated as mitotic chromosome condensation. Cells also chose to repair damage revealed in anaphase without an arrest, "on the fly" as it were, with DNA breaks, merotelic mis-attachments, and residual catenations being continuously healed/eliminated throughout anaphase.

Depending on how the chromosomal bridges are generated, there could be more specific biological reasons for uncoupling certain processes from checkpoint controls. For example, cleavage of cohesin and removal of all inter-chromatid catenations may be unrecognizable by checkpoints because residual interlocking of sister chromatids is actually needed for proper timing of anaphase. This idea is supported by the fact that late-segregating rDNA loci in yeast do segregate faster [40] and the genome becomes less stable [95] when tandem rDNA repeats are converted into episomes. Unfortunately, we still know very little about the proper control of anaphase length.

In the case of merotelic attachments, it is clear that cells pay a price for kinetochore complexity. While budding yeast, with a single microtubule to kinetochore attachment, have no problem with merotely in mitosis, even though molecularly-related syntely is possible, higher cells have to deal with this challenge proportionally to the size of kinetochore, which relates to the number of microtubule-binding modules and their spacing (Fig. 5). Once again, it is not known why merotelic attachments are incompletely, i.e. poorly (!), corrected in metaphase. One logical explanation, is that 1) the stochastic nature of this repair, involving cycles of kinetochore uncoupling and re-coupling to the spindle, and 2) the readouts based on shifts in tension and on the inner centromere to kinetochore distance are in principle unable to remove all mis-attachments because they constantly reemerge as a part of the repairing process itself. While directing sister kinetochores away from each other is not a big problem with condensed sister centromeres and compact kinetochores facing outwards (Fig. 5B), this "vector" approach to merotely correction fails upon aberrant centromere decondensation, especially in human and *C. elegans *cells with large kinetochores.

With respect to under-replication, it is extremely puzzling that ongoing replication apparently does not always send a "stop anaphase" signal. One can envision several rationales for this. One is a requirement for reparative DNA synthesis to occur throughout the cell cycle. Indeed, site-specific DNA replication is required to repair some internally generated damage after the completion of S-phase, such as during erasing of DNA methylation [96,97], or potentially during removal of DNA-bound proteins that are not dissociated in some tissues during DNA synthesis [98]. A more global explanation would be that the final moments of replication can overlap with mitosis by a structural default (such as centromere replication or condensin targeting). It is nevertheless clear that the end of replication process has a yet underappreciated role in cell cycle regulation. Ongoing experiments should enable bringing new insights to understanding this fascinating phenomenon.

## Competing interests

The author declares that he has no competing interests.
